# Zinc Preconditioning Provides Cytoprotection following Iodinated Contrast Media Exposure in In Vitro Models

**DOI:** 10.1155/2021/6686803

**Published:** 2021-02-17

**Authors:** Marlon Perera, Joseph Ischia, Damien Bolton, Arthur Shulkes, Graham S. Baldwin, Oneel Patel

**Affiliations:** ^1^University of Melbourne Department of Surgery, Austin Health, Melbourne, Victoria, Australia; ^2^Olivia Newton-John Cancer Research Institute, Austin Hospital, Heidelberg, Victoria, Australia

## Abstract

**Methods:**

Normal human proximal renal kidney cells (HK-2) were preconditioned with either increasing doses of ZnCl_2_ or control. Following this preconditioning, cells were exposed to increasing concentrations of Iohexol 300 mg I_2_/ml for four hours. Key outcome measures included cell survival (MTT colorimetric assay) and ROS generation (H_2_DCFDA fluorescence assay).

**Results:**

Contrast media induced a dose-dependent reduction in survival of HK-2 cells. Compared to control, contrast media at 150, 225, and 300 mg I_2_/ml resulted in 69.5% (SD 8.8%), 37.3% (SD 4.8%), and 4.8% (SD 6.6%) cell survival, respectively (*p* < 0.001). Preconditioning with 37.5 *μ*M and 50 *μ*M ZnCl_2_ increased cell survival by 173% (SD 27.8%) (*p* < 0.001) and 219% (SD 32.2%) (*p* < 0.001), respectively, compared to control preconditioning. Zinc preconditioning resulted in a reduction of ROS generation. Zinc pre-conditioning with 37.5 *μ*M *μ*M ZnCl_2_ reduced ROS generation by 46% (*p* < 0.001) compared to control pre-conditioning.

**Conclusions:**

Zinc preconditioning reduces oxidative stress following exposure to radiographic contrast media which in turn results in increased survival of renal cells. Translation of this *in vitro* finding in animal models will lay the foundation for future use of zinc preconditioning against contrast induced nephropathy.

## 1. Introduction

Advances in the availability, utility, cost, and the safety of computerised tomography (CT) have corresponded with its increasing use globally [1, 2]. Contrast media (CM) is frequently administered at the time of CT to improve diagnostics. Contrast-induced nephropathy (CIN) is an iatrogenic disease occurring following the parenteral administration of CM and is objectively defined by the development of acute renal failure (ARF) within 24–72 hours of CM exposure. ARF is characterised by either the increase of serum creatinine (by 25%) or decrease of the estimated glomerular filtration rate (to 30–60 mL/min).

The complex pathophysiology relating to the development of CIN has been well documented. Broadly speaking, CIN occurs as a result of two principal pathways that relate to both the cytotoxic properties and the osmolar properties of the CM. The cytotoxic pathway of CIN results in direct cellular injury and additionally mediates locoregional alterations in renal medullary perfusion and results in renal medullary hypoxia. The osmolar properties of the CM result in osmotic nephrosis and further augment medullary hypoxia. As such, the key change that results in CIN is the process of renal medullary hypoxia [3–6]. The outer medulla of the kidney is particularly vulnerable to hypoxia due to high oxygen requirements from salt absorption in the Loop of Henle's thick limb, the S3 segment of the proximal renal tubules. Hypoxia results in production of reactive oxygen species (ROS) and results in cellular injury. Zinc is a critical physiologic nutrient that has multiple properties. Intracellularly, zinc appears to provide an integral role in homeostasis of ROS. Indeed, an increase in ROS levels contributes to a rapid increase in intracellularly free-zinc due to the mobilization from intracellular zinc stores.

The cytotoxic effects of contrast on renal cells may have been effectively assessed in *in vitro* models. Specifically, several groups have outlined the reno-protective effects of various agents on HK-2 cell lines [7–14]. The aim of the current study is to assess the outcomes of zinc pre-conditioning on HK-2 renal cell lines following contrast administration.

## 2. Methods

### 2.1. Culture Conditions and Reagents

Human Kidney 2 (HK-2) cells (CRL-2190™) were obtained from American Type Culture Collection (ATCC) (Manassas, VA, Unite States of America). HK-2 cells are derived from normal human male renal cortex and proximal tubules. Cells are immortalized by transduction with human papillomavirus 16 (HPV-16) E6/E7 genes [15]. HK2 cells were cultured in 75 cc flasks in the aforementioned HK-2 culture media (DMEM : F12, supplemented with HEPES, Penicillin, Streptomycin and FBS). Cells were maintained at 37°C in a humidified incubator with 95% air and 5% CO_2_.

A single contrast medium was utilized for all studies. Iohexol 300 mg I_2_/mL (Omnipaque 300, GE Healthcare, Buckinghamshire, United Kingdom) has the formulation N, N′-bis (2, 3-dihydroxypropyl)-5-[N (2,3-dihydroxypropyl)acetamido]-2,4,6-triiodoisophthalamide. This represents a tri-iodinated, non-ionic hyperosmolar contrast agent with a molecular weight of 821.14. Additional components to Omnipaque include sodium calcium edetate, hydrochloric acid, and water for injection. As per the manufacturers' specification, Omnipaque 300 exhibits the following chemical characteristics: osmolality = 0.64 Osm/kg H_2_O or 640 mOsm/kg H_2_O, viscosity 11.6 mPas at 20°C and 6.1 at 37°C.

### 2.2. Experimental Design

The study was divided into three principal experiments:

- To determine the effect of increasing concentrations of contrast media exposure (10 mg I_2_/mL, 20 mg I_2_/mL, 40 mg I_2_/mL, 60 mg I_2_/mL, 80 mg I_2_/mL, 150 mg I_2_/mL, or 300 mg I_2_/mL), for four hours on cytotoxicity and cell viability.
- To determine the effect of contrast media exposure on the production of ROS.
- To assess the effect of Zinc preconditioning on CM-induced apoptosis and ROS levels. Cells were preconditioned with increasing concentrations of zinc for 4 or 15 hours.

### 2.3. Measurement of Cell Viability

HK-2 cells were cultured and at 80% confluence, cells were split and plated in a 6-well plate. 250,000 cells per well were plated in 1500 *μ*L of HK-2 serum medium and cultured for 12 hours. HK2 cells were then pre-conditioned as required for 4 or 15 hours. Following preconditioning, the preconditioning substrate was removed and replaced with the CM at varying concentrations (10 mg I_2_/mL, 20 mg I_2_/mL, 40 mg I_2_/mL, 60 mg I_2_/mL, 80 mg I_2_/mL, 150 mg I_2_/mL, or 300 mg I_2_/mL), diluted in HBSS. Cells were exposed to CM for 4 hours, and then the contrast solution was removed and replaced with HK-2 serum-free media.

To assess cell survival and viability, the proliferative measure utilized was the MTT assay (3-(4,5-dimethylthiazol-2-yl)-2,5-diphenyltetrazolium bromide). MTT is a colorimetric assay assessing the cellular metabolic activity. Specifically, metabolically active cells may be quantified by the activity of NADPH dependent cellular oxidoreductase enzyme function. Active cells reduce the 3-(4,5-dimethylthiazol-2-yl)-2,5-diphenyltetrazolium bromide dye to an insoluble formazan—which is purple in colour. A formazan solubilizing solution is added (isopropanol) and the colour may be assessed at a certain wavelength [16, 17].

Following recovery, MTT tracer (30 *μ*L) was added and left for 60 minutes. At this time, formazin solubilizing solution was added. 100 *μ*L of the fluid was removed and added to a 96-well plate for reading. Each reading was performed in triplicate using FLUOstar OPTIMA (BMG Labtech, Ortenberg, Germany) photocolorimetric and fluorescence reader. Data was collected using Optima v2.10R2 (BMG Labtech, Ortenberg, Germany) utilizing photocolorimetric assessment with absorbance filters of 570 nm and 620 nm. A latter reading of 620 nm was utilized as a reference standard and was subtracted from the former reading. The resulting value was regarded as the luminescence unit for the respective assay.

### 2.4. Measurement of Reactive Oxygen Species

HK-2 cells were cultured and at 80% confluence cells were split and plated in a 96-well black plate. Typically, 15,000 to 17,000 cells per wells were plated in 100 *μ*L of HK-2 serum medium and cultured for 12 hours. HK-2 cells were exposed to pre-conditioning as required. HK-2 serum medium was removed and replaced with the preconditioning substrate for four or 15 hours. Following this period, preconditioning substrate was removed and replaced with the contrast substrate. Iohexol 300 mg I_2_/mL was diluted to pre-specified concentrations, typically 10 mg I_2_/mL, 20 mg I_2_/mL, 40 mg I_2_/mL, 60 mg I_2_/mL, 80 mg I_2_/mL, 150 mg I_2_/mL, or 300 mg I_2_/mL. Contrast medium was diluted in HBSS. Cells were maintained in the contrast solution for 2 or 4 hours depending on the study conditions. After this period, CM was removed and washed with HBSS and the ROS probe was introduced.

DCFDA was diluted to 1 : 1000 and protected from light at all times. 50 *μ*L of the diluted DCFDA solution (diluted 1 : 1000) was added to each well and maintained for 30 mins. At this point, residual tracer was removed and 100 *μ*L of HBSS was placed in each well for assessment. The 96-well plate was assessed using FLUOstar OPTIMA (BMG Labtech, Ortenberg, Germany) photocolorimetric and fluorescence reader. Data was collected using Optima v2.10R2 (BMG Labtech, Ortenberg, Germany) utilizing fluorescence assessment with an excitation filter of 485 nm and an emission filter of 520 nm.

Generation of ROS as measured by DCFDA is dependent on cell numbers. Accordingly, it is recommended that standardization of ROS levels should be performed as a function of cell numbers [18]. While there is no universal method for quantifying cell number, it may be performed by quantifying protein level or cell viability as measured by MTT on 96-well plates concurrently. MTT assay was performed at the same time-point as the corresponding DCFDA assay.

### 2.5. Statistical Analysis

Data were entered in an Excel 2016 spreadsheet (Microsoft, Redmond, WA, USA). Data were transferred to GraphPad Prism (version 7.0, GraphPad Software, La Jolla, CA, United States of America) for data analysis.

For DCFDA and MTT, raw florescence and luminescence units were recorded, respectively. The change in florescence or luminescence values was determined by subtracting the value at a particular contrast dose from its respective control and was expressed as Delta luminescence or Delta florescence. The proportional change was calculated by representing the test value over the mean of the control value and was expressed as % Delta luminescence of % Delta florescence.

For DCFDA assays, raw luminescence units were recorded as per the output of the photocolorimetric reader utilized. A dose-dependent quenching effect between the DCFDA probe and contrast media was observed. The degree of quenching for a particular does was quantified and expressed as a coefficient to allow for correction. Corrections for the degree of quenching were performed in Excel by multiplying the luminescence units by the predetermined quenching coefficient at the particular contrast dose.

Data are presented as means ± the standard error of the mean (SEM) unless otherwise stated. For normally distributed data, Student's *t*-test was used to determine statistical significance. For non-parametric data, the Mann–Whitney rank sum test was utilized. For multiple comparisons, the one-way ANOVA followed by the Bonferroni correction was performed.

## 3. Results

### 3.1. Iodinated Contrast Media Decrease Cell Viability

**Figure 1:**
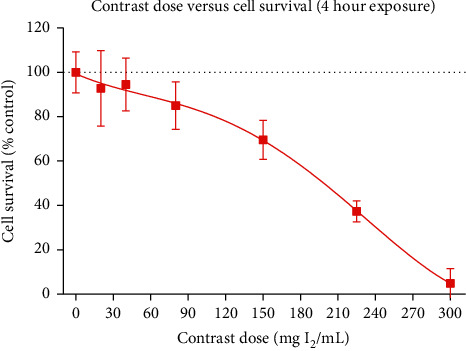
Dose-dependency of cell survival (MTT) compared to control (no contrast).

To determine the optimal dosing scheme for contrast media, a dose-dependency association between HK-2 cells and four-hour exposure of increasing contrast media concentration was performed. Compared to control (0 mg I_2_/mL of contrast media), cell survival as measured on MTT colorimetric assay reduced with increasing doses. Dose response exhibited a non-linear relationship overall. Contrast media at a dose of 150 mg I_2_/mL resulted in 69.5% cell survival. This number was reduced to 37.3% and 4.8% with 225 and 300 mg I_2_/mL (Figure 1).

### 3.2. Iodinated Contrast Media Increase ROS Production

Cells were exposed to increasing concentrations of contrast media (10, 20, 40, 80, 150, 225, and 300 mg I_2_/mL) and a correction for the quenching effect of contrast media was applied. Corresponding MTT assay was performed in a 96-well plate concurrently to account for reductions in cell numbers. Following correction for reduced cell numbers (MTT absorbance), a significant increase in fluorescence was observed with increasing concentrations of CM. Exposure of HK2 cells to 300 mg I_2_/mL resulted in a 10-fold increase in DCFDA florescence compared to controls.

### 3.3. Zinc Preconditioning Has Protective Effect on Cell Viability and Production of ROS

**Figure 2:**
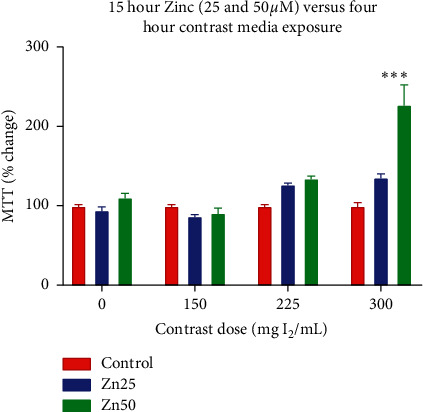
Cell viability following increasing concentration of CM exposure following control and preconditioning with 25 *μ*M and 50 *μ*M.

Cells were preconditioned for 15 hours with zinc (25 *μ*M and 50 *μ*M) and treated with contrast media (150, 225, and 300 mg I_2_/mL) for four hours. Compared to the control group, 15-hour preconditioning did not provide reproducible, statistical significant improvements in cell-survival when exposed to contrast media at 150 and 225 mg I_2_/ml. However, at a dose of 300 mg I_2_/ml, Zn preconditioning with 25 *μ*M and 50 *μ*M resulted in a 135.9% and 227.3% increase in cell survival (*p* < 0.0001, one-way ANOVA) (Figure 2).

**Figure 3:**
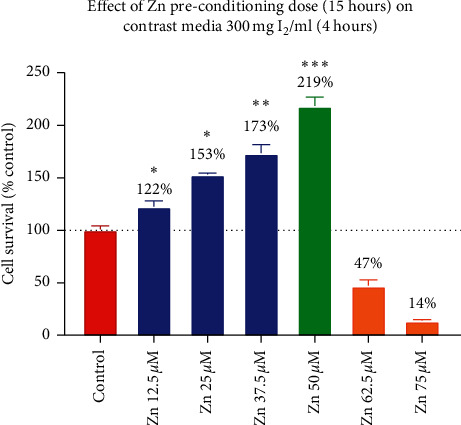
Cell viability following dose escalation for Zinc preconditioning with 12.5 *μ*M, 25 *μ*M, 37.5 *μ*M, 50 *μ*M, 62.5 *μ*M, and 75 *μ*M.

Zinc dose escalation was performed to determine the optimal dosing required for preconditioning. Cells were preconditioned with zinc chloride doses ranging between 12.5 *μ*M and 75 *μ*M. Between 12.5 *μ*M and 50 *μ*M, 15 hours of zinc preconditioning resulted in increased cell survival compared to control preconditioning. Between these ranges, cell survival increased incrementally from 122% (95% CI: 110.8–134.4%, *p*=0.002) to 219% (95% CI: 200.7–236.4%, *p* < 0.001) with 12.5 *μ*M and 50 *μ*M, respectively. Preconditioning with zinc chloride of concentrations greater than 50 *μ*M resulted in a reduction of cell survival when compared to control (Figure 3).

**Figure 4:**
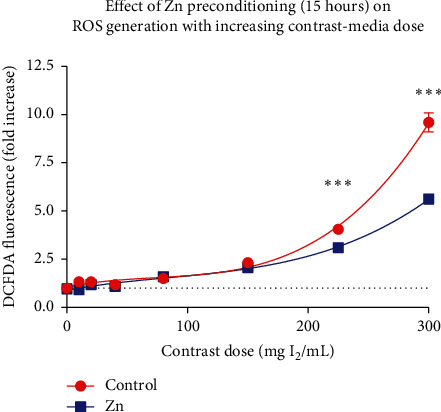
Zinc preconditioning (15 hours) vs. control prior to contrast media exposure on DCFDA fluorescence.

Regarding ROS, cells were preconditioned with zinc chloride 50 *μ*M for 15 hours and exposed to increasing concentrations of contrast media (10, 20, 40, 80, 150, 225, and 300 mg I_2_/mL) and a correction for the quenching effect of contrast media was applied. Corresponding MTT assay was performed in a 96-well plate concurrently to account for reductions in cell numbers. Zinc preconditioning resulted in a reduction in DCFDA florescence. Statistical significance was reached in contrast concentrations of 150 (26.0% reduction, *p*=0.009, Student's *t*-test), 225 (96.1% reduction, *p* < 0.0001, Student's *t*-test), and 300 mg I_2_/mL (397.7% reduction, *p* < 0.0001, Student's *t*-test) (Figure 4).

## 4. Discussion

The results from our *in vivo* model for contrast-induced nephropathy resulted in several key findings. Most pertinently, in-principal zinc chloride preconditioning results in improved cell survival and reduced ROS generation when compared to control preconditioning. Zinc chloride preconditioning demonstrated a linear dose-dependent response up to an optimal threshold concentration.

In our cellular model of CIN, we were able to reliably demonstrate loss of cell viability following exposure to CM. The pathophysiological process of CIN has been well studied, with hypothesized cytotoxic pathway and ischaemic pathways. Our study demonstrated a dose-dependent response of cell viability with increasing doses of CM (Figure 1). Specifically, cell viability following exposure with CM at concentrations of 150, 225, and 300 mg I_2_/mL was 69.5%, 37.3%, and 4.8%. Such findings have been demonstrated on previously published HK2 models using similar MTT methods to assess cell viability [10, 11, 13]. Peng et al. similarly identified a dose-dependent response with cell viability decreasing progressively from 100% to 12% following exposures of 80 mg I/ml over 16 hours [11]. Yao et al. reported a similar trend, however to a lesser magnitude. Following exposure for 24 hours with increasing concentrations of CM up to 200 mg I/ml, cell viability dropped progressively to 77% [13]. While the dose-dependent response of cell viability is reproducible with comparable series, there is some variation in magnitude of this change.

The precise mechanism of the cytotoxic pathway for reduced cell viability and apoptosis is likely multifactorial. The findings from the current study support the likely role of reactive oxygen species in this pathway. We demonstrated a consistent increase in DCFDA fluorescence, an accepted surrogate for ROS levels, following increasing concentrations of CM. Intuitively, increasing levels of ROS provides a higher degree of oxidative stress on HK2 cells and results in compromised cell viability. Several series assessing HK2 models have also assessed ROS production using the aforementioned DCFDA probe [8, 12, 14]. Despite its widespread use, the DCFDA probe is an indirect measure of ROS. The DCFDA probe enters cells and is de-esterified and oxidized to a fluorescent DCF (2′,7′-dichloroflourescein)—a process a result of intra-cellular ROS [19]. The mechanism by which ROS results in cytotoxicity is likely due to the resulting production of O_2_^−^, H_2_O_2_, and ^.^OH which results in direct cellular and DNA injury. Previous comparable series have directly quantified DNA damage by means of DNA fragmentation assays [13]. Regardless of the precise mechanism, we have demonstrated that an increase in ROS following contrast exposure is intricately related to cell viability with increasing concentrations. We demonstrated that preconditioning with Zn prior to CM exposure (of 300 mg I_2_/ml) results in a 50% reduction in ROS production based on DCFDA assays. To further demonstrate this, we identified that zinc has a dose-dependent response with respect to cell viability. Specifically, improved cell viability increased progressively with increasing concentrations of zinc preconditioning, up to a threshold point of 50 *μ*M (*p* < 0.01). After this point, cell viability was greatly reduced, likely a result of impaired intracellular homeostatic mechanisms and altered osmolarity.

Current evidence supports the notion that zinc provides an integral role in homeostasis of ROS. This is evidenced by the fact that increases in ROS levels are followed by rapid increases in intracellularly free-zinc due to the mobilization from intracellular zinc stores and release from metallothionein [20, 21]. Zinc limits ROS production and the sequelae by several mechanisms as outlined previously. Firstly, the release of zinc by metallothionein frees metallothionein, which has antioxidant properties due to the high cysteine content which scavenges ^.^OH [22]. Secondly, zinc reduces other cations, such as Fe^2+^ and Cu^2+^ ions, on the cell membrane and reduces the production of ^–^OH from H_2_O_2_ that these metals physiologically catalyze. Thirdly, zinc upregulates production and activation of antioxidant proteins (such as glutathione, catalase, and superoxide dismutase) [23] and downregulates oxidant-promoting enzymes (such as inducible nitric acid synthase and NADPH enzyme). The benefit of zinc on ROS modulation has been demonstrated in in vitro, animal and models [24, 25]. On a cellular basis, in vitro cell culture models have demonstrated reproducible improvements in ROS markers including lipid peroxidation productions, DNA oxidation products, and inflammatory cytokines. These changes have been demonstrated in various cell lines including promyelocytic leukemia cells, human aortic endothelial cells, and human isolated peripheral blood mononuclear cells [24, 26, 27]. These findings have been further extrapolated to small animal models in rats [28–31]. In human models, various studies have demonstrated the effect of zinc supplementation and zinc deficiency on lipid peroxidation and ROS parameters. For example, Roussel et al. demonstrated that zinc supplementation was correlated with reduced levels of plasma thiobarbituric acid reactive substances and other ROS parameters in diabetic patients [32]. These findings have been corroborated in comparable studies [33, 34]. These models lay the foundations for assessing the potential therapeutic effect of zinc preconditioning on various forms of cellular injury instigated by ROS.

There are several limitations to the current in vitro model of CIN. Firstly, the current model only utilizes a single cell line (renal tubular cells). Further assessment using alternate cell lines may provide additional information regarding the reproducibility of the current findings. Additionally, only one measure of ROS was utilized in this model. While DCFDA is a well-established measure of ROS production, additional measures including those mediated through lipid peroxidation may be of utility to confirm this increase in ROS. The precise mechanism by which zinc preconditioning results in improved cell viability and reduction ROS is of importance and represents a direction for future research. Further, extrapolation of this in vitro model to a small animal in vivo model is planned in the future.

## 5. Conclusion

In conclusion, our in vitro model of CIN resulted in reproducible, dose-dependent increase in ROS production and compromised cell viability. We have demonstrated that zinc preconditioning provides protection against ROS production and resulting cell viability. The cytoprotective effects of zinc preconditioning further demonstrated a dose-dependent response. Further in vivo trials are required to further determine the clinical implications of these findings.

## Data Availability

The data used in the study are available on request to the corresponding author (marlonlperera@gmail.com).
